# Powdered activated carbon (PAC)-assisted peroxymonosulfate activation for efficient urea elimination in ultrapure water production from reclaimed water

**DOI:** 10.1038/s41598-024-55414-w

**Published:** 2024-02-26

**Authors:** Chaelin Kim, Heeji Yoo, Gyubin Lee, Hye-Jin Hong

**Affiliations:** Department of Environmental Engineering, Chungbuk National University, Chungdaero 1, Chungbuk, SeowonGu, Cheongju, 28644 Republic of Korea

**Keywords:** Chemical engineering, Pollution remediation

## Abstract

Urea is a problematic pollutant in reclaimed water for ultrapure water (UPW) production. The sulfate radical-based advanced oxidation process (SR-AOP) has been recognized as an effective method for urea degradation. However, conventional metal-based catalysts for peroxymonosulfate (PMS) activation are unsuitable for UPW production due to issues related to metal ion leaching. In this study, the use of powdered activated carbon (PAC) was investigated for the removal of urea from reclaimed water. The PAC exhibited a high degree of defects (I_*D*_/I_*G*_ = 1.709) and various surface oxygen functional groups (C–OH, C=O, and C–O), which greatly enhanced its catalytic capability. The PAC significantly facilitated PMS activation in the PMS + PAC system, leading to the complete urea decomposition. The PMS + PAC system demonstrated excellent urea removal efficiency within a wide pH range, except for pH < 3. Among the various anions present, the CO_3_^2−^ and PO_4_^3−^ inhibited urea degradation, while the coexistence of Cl^−^ promoted urea removal. Furthermore, the feasibility test was evaluated using actual reclaimed water. The quenching test revealed that SO_4_^−^*·*, *·*OH, and O_2_^−^*·* played crucial roles in the degradation of urea in the PAC-assisted SR-AOP. The oxygen functional groups (C–OH and O–C=O) and defect sites of PAC clearly contributed to PMS activation.

## Introduction

The demand for ultrapure water (UPW) is increasing due to advancements in high-technology industries such as semiconductor manufacturing, display production, and pharmaceuticals^1^. The UPW refers to water with extremely low total dissolved solids (TDS) levels, typically less than 5.0 ppb^2^. Tap water is commonly used as the source water for UPW production, but it undergoes additional physicochemical treatments such as ion exchange, reverse osmosis (RO), UV-oxidation, and decarboxylation to remove trace elements present in the source water^3^. A huge amount of source water is required to produce UPW, but the source of tap water is limited, and it should be supplied first to people for essential daily activities. To achieve both economic viability and water resource sustainability, there is a growing interest in utilizing reclaimed water, which refers to treated wastewater discharged after undergoing a wastewater treatment process, as an alternative to tap water.

Urea is a common contaminant found in reclaimed water, and its presence poses challenges in producing high-quality UPW due to the difficulty of removing urea from reclaimed water by conventional UPW production process^4^. Traditional UPW production processes, such as activated carbon adsorption or reverse osmosis (RO), face difficulties in effectively removing urea due to its low molecular weight and nonpolar nature^5,6^. Also, the urea is hardly degraded by UV oxidation due to its strong N-bonds^7^. Additionally, its non-ionic nature makes it challenging to remove using ion exchange resins. Some studies have observed only about 20–50% efficiency in urea removal using RO membranes^4^. Also, the urea removal efficiency via UV light has been reported to be less than 10%^6^. Indeed, there have been cases where the TOC in UPW measured high in semiconductor factories due to incomplete impurity removal^8^. Although ongoing research focuses on the removal of organic contaminants from reclaimed water, there has been limited specific research on urea removal. Therefore, there is a need for the effective removal of urea in reclaimed water to produce UPW.

In recent years, there has been significant research focused on sulfate radical-based advanced oxidation processes (SR-AOPs) for the degradation of persistent organic contaminants. The sulfate radical (SO_4_^−^∙) is a strong oxidant with a high redox potential and a long half-life (2.6–3.1 V, 30–40 μs) compared to hydroxyl radical (∙OH, 1.8–2.7 V, 10^–3^ μs) used in conventional advanced oxidation processes (AOPs)^9^. One notable advantage of the sulfate radical is its high affinity to electron-donating groups such as amine (–NH_3_), which is present in urea^10^. This characteristic suggests that SR-AOP is highly efficient in degrading trace amounts of urea present in reclaimed water.

Sulfate radicals can be generated from peroxymonosulfate (PMS) or peroxydisulfate (PDS) through various activation methods, including alkali^11^, UV^12^, ultrasound^13^, and transition metal catalysts^14^. Among these methods, alkali activation poses challenges for UPW production due to the neutralization process caused by pH changes. UV and ultrasound activation methods are relatively inefficient and consume significant amounts of energy. The most efficient method for generating SO_4_^−^· is through metal catalyst activation. However, this approach carries the potential risk of secondary pollution by the leaching of metals^15^. Therefore, it is not appropriate to use this method for treating reclaimed water in UPW production.

To address the issue of metal leaching of metal catalysts, we explored the potential use of powdered activated carbon (PAC) as a non-metal catalyst for the degradation of urea in reclaimed water. PAC has garnered attention due to its extensive surface area and porosity, resulting in strong adsorption capacity and high catalytic activity. While extensive studies have been conducted on the activation of PMS or PDS using activated carbon (AC) to remove organic pollutants^16–18^, there has been limited research on employing this method for the elimination of urea in the production of ultrapure water.

Here in, we aimed to assess the efficacy of powdered activated carbon (PAC) as a catalyst for promoting peroxymonosulfate (PMS) activation in effectively removing urea. Moreover, we examined the physicochemical changes in PAC before and after the urea degradation reaction to elucidate the activation mechanism of PMS and the underlying mechanism of urea degradation. The investigation encompassed the following key aspects: (i) analysis of the physicochemical properties of PAC as a catalyst for PMS activation; (ii) conducting urea degradation studies systematically, under various conditions including the dosage test of PAC and PMS, initial pH, water matrix, and evaluating the feasibility of urea degradation in actual reclaimed water; (iii) finally, discovering the urea degradation mechanism in SR-AOP using PAC as a catalyst.

## Results

### Characterization of PAC

**Figure 1:**
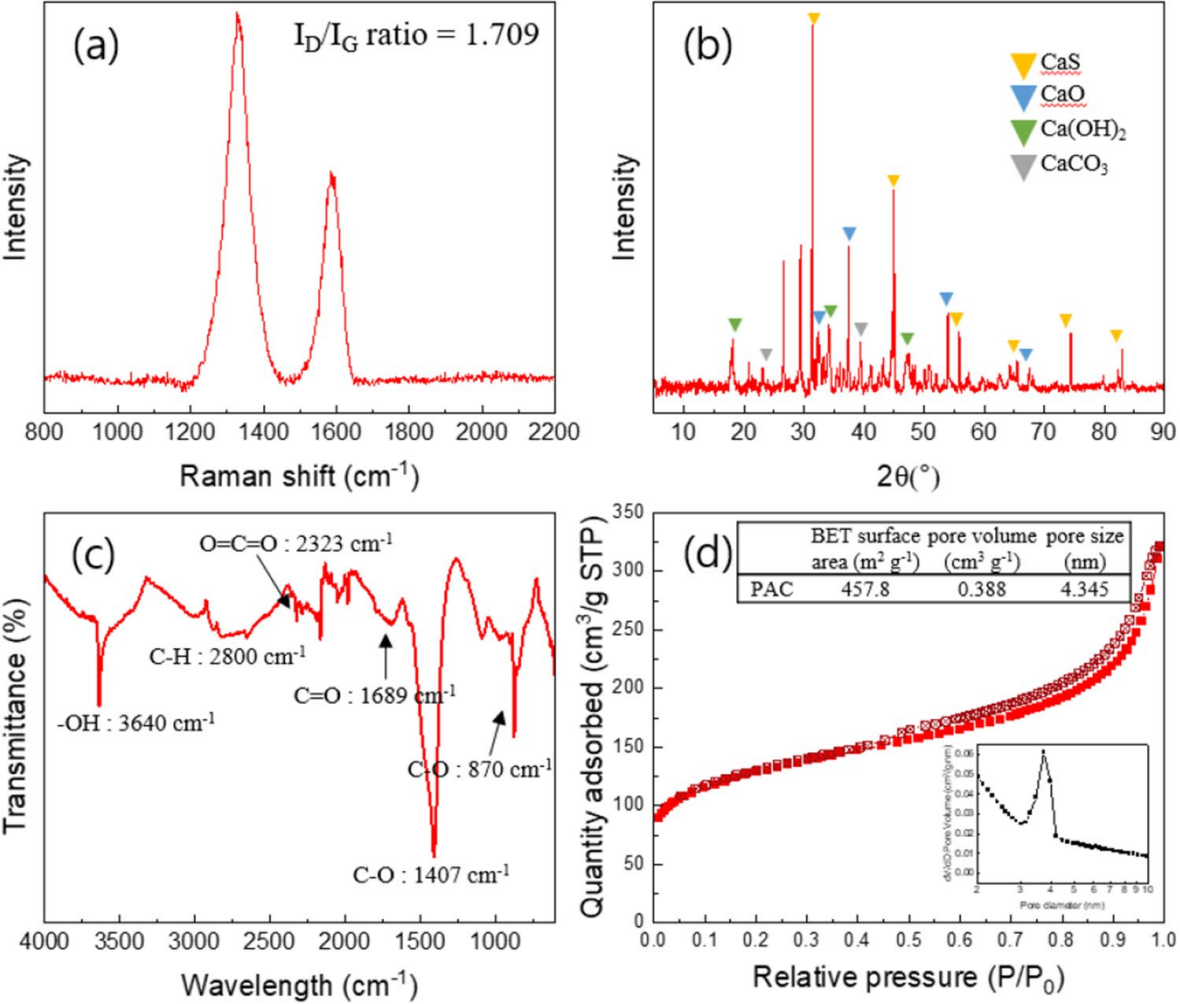
(**a**) Raman spectrum, (**b**) XRD pattern, (**c**) FTIR spectrum, and (**d**) N_2_ adsorption–desorption isotherm of the PAC.

Raman spectrum of the PAC was shown in Fig. 1a. As it is known, carbon-based materials have two crucial characteristic peaks: D peak (1320–1350 cm^−1^) and G peak (1570–1585 cm^−1^)^19^. The D peak observed at 1330 cm^−1^ indicates defects in the graphitic structure, resulting from out-of-plane vibrations. The G peak observed at 1585 cm^−1^ arises from in-plane vibrations of the C–C bond within the *sp*^2^ orbital, indicating a graphitic structure^20^. The I_D_/I_G_ ratio, which represents the intensity ratio of the D and G peaks, provides direct evidence of the degree of defects in the graphitic structure^21^. In the case of the PAC, the I_D_/I_G_ ratio was measured to be 1.709, indicating a relatively high degree of defects. It is widely known that defect sites in carbon-based materials can facilitate electron transfer between the catalyst and PMS^19^.

XRD pattern of the PAC was shown in Fig. 1b. The observed peaks at 2θ = 31.4°, 45.0°, 55.8°, 65.4°, and 74.4° corresponded to the (200), (220), (222), (400), and (420) crystal planes of the calcium sulfide (CaS)^22^. Also, peaks at 32.2°, 37.4°, 53.9°, and 67.5° were ascribed to the (111), (200), (220), and (222) of the calcium oxide (CaO)^23^. Furthermore, the peaks at 18.0°, 34.0°, and 47.0° were observed, corresponding to the (001), (101), and (102) of the calcium hydroxide (Ca(OH)_2_) and peaks at 23.0°, 29.3°, and 39.4° corresponded to the (012), (104), and (113) of the calcium carbonate (CaCO_3_)^24,25^. Based on this XRD analysis, it is evident that the PAC contains various crystalline calcium compounds as impurities.

To determine the surface functional groups of the PAC, FTIR analysis was conducted (Fig. 1c). The peaks at 870 and 1407 cm^−1^ were assigned to C–O stretching vibrations^26^. In addition, 1689 and 2323 cm^−1^ belonged to the vibrations of C=O and O=C=O, respectively^27^. The C–H vibration was observed at around 2800 cm^−1^ and a sharp peak located at around 3640 cm^−1^ was associated with the characteristic stretching of –OH^28^. Previous studies have indicated that the abundance of surface oxygen-containing groups (OCGs) in catalysts enhances electrical conductivity and contributes to the activation of PMS^29^.

The N_2_ adsorption–desorption isotherm of the PAC was shown in Fig. 1d. The BET specific surface area of the PAC was 457.8 m^2^g^−1^, and the pore volume was measured as 0.388 cm^3^g^−1^. The BET analysis revealed that the PAC is a mesoporous material (2–50 nm), which has a 4.34 nm average pore size. Mesoporous materials are well-known for their high specific surface areas, which contribute to their remarkable adsorption capacity and catalytic properties^30^. Furthermore, the large specific surface area offers several advantages such as promoting of electron transfer process and catalytic activity^31^.

### Degradation test of urea by PAC assisted SR-AOP

**Figure 2:**
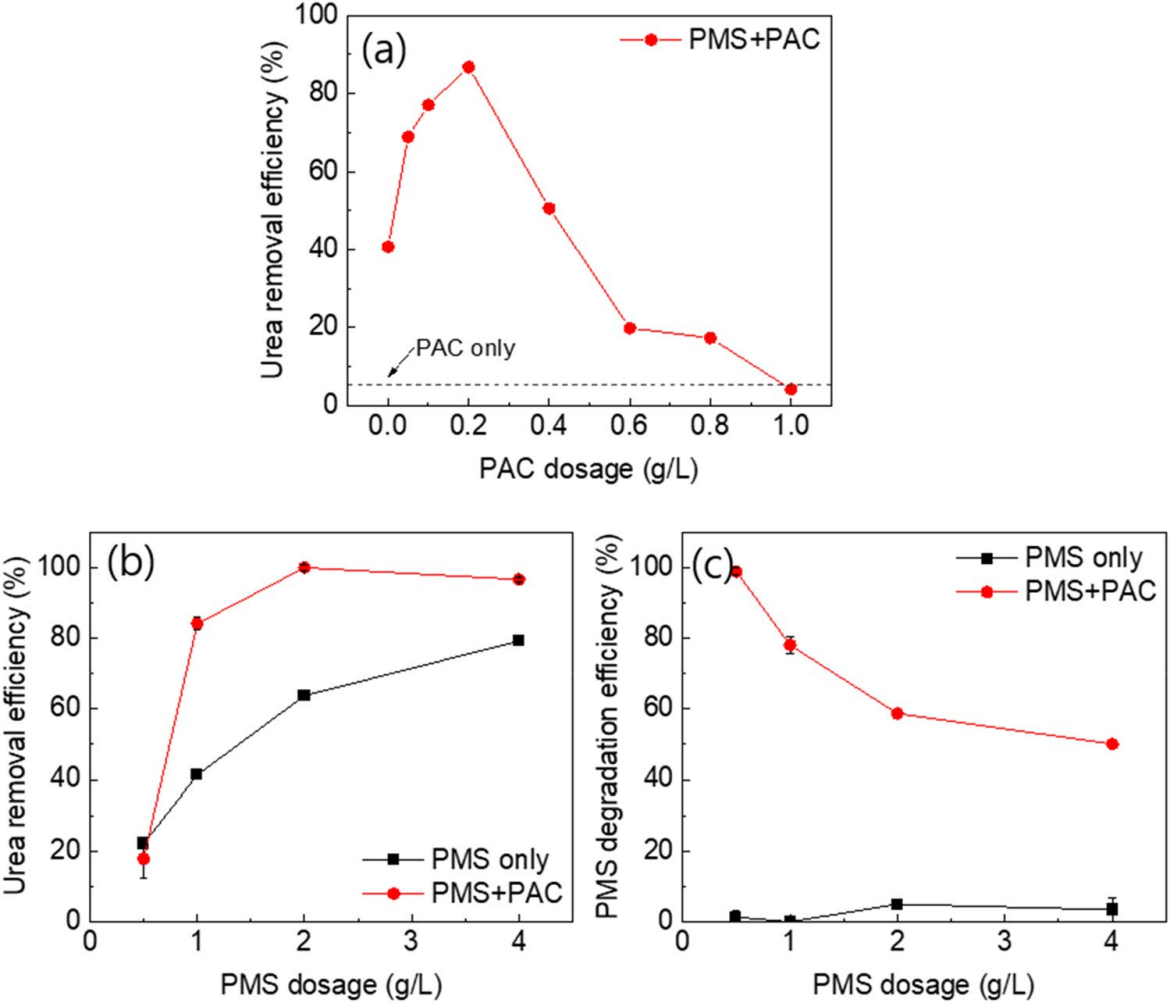
(**a**) The dosage test of PAC (**b**) and PMS, (**c**) and the degradation efficiency of PMS.

To determine the optimal conditions for the PAC assisted SR-AOP (PMS + PAC system), we investigated the effect of PAC and PMS dosage. For comparison, we also evaluated the urea degradation efficiency in the PAC only system (without PMS) and the PMS only system (without PAC). As shown in Fig. 2a, the removal efficiency of urea in the PAC only system (PAC = 1.0 g/L) was found to be less than 5.0%. It results in the adsorption of urea onto the PAC, even in the absence of PMS. However, due to the low affinity of urea for the PAC, only a minimal amount of urea was adsorbed onto the PAC. In contrast, in the PMS + PAC system, the removal efficiency increased proportionally with the PAC dosage, ranging from 0.05 to 0.2 g/L. The highest removal efficiency (86.8%) was observed at the PAC dosage of 0.2 g/L. This result could be attributed to the activation of PMS and the formation of ROS due to the introduction of PAC (Eq. 1). This implies the occurrence of synergistic effects and the involvement of additional mechanisms in the removal of urea by the PMS + PAC system. The increase in PAC dosage led to an increase in PMS activation sites, thereby promoting the formation of ROS^32^. However, when the PAC dosage exceeded 0.4 g/L, the excessive dosage of PAC stimulated the activity of PMS, resulting in an immoderate formation of ROS and a subsequent decline in removal efficiency. This can be attributed to the fact that excessive free radicals may scavenge themselves or convert into other species with weaker redox potentials through reactions with S_2_O_8_^2−^ (Eqs. 2–4)^33,34^. In summary, the results highlight the importance of optimizing the PAC and PMS dosage for efficient urea degradation. The findings suggest that the synergistic effect between PMS and PAC, plays a crucial role in achieving high urea removal efficiency. To evaluate the catalytic performance of PAC for SR-AOP for urea degradation, we compared the catalytic activities of other carbon-based catalysts, including granular activated carbon (GAC), graphene oxide (GO), single-walled carbon nanotube (SWCNT), and multi-walled carbon nanotube (MWCNT). Among these carbon-based materials, PAC exhibited the highest catalytic activity for urea degradation (Table S2). This superiority is anticipated to be due to its large surface area, high density of oxygen-containing groups, and high I_D_/I_G_ ratio. This result clearly demonstrated that PAC is a highly effective carbon-based catalyst for urea degradation in SR-AOP.

1 $$ {\text{HSO}}_{5}^{{{ } - }} { } + e^{ - } { } \to { }SO_{4}^{ - } \cdot + \cdot OH $$

2 $$ {\text{SO}}_{4}^{{{ } - }} \cdot { } + {\text{ SO}}_{4}^{{{ } - }} \cdot { } \to {\text{ S}}_{2} {\text{O}}_{8}^{{{ }2 - }} $$

3 $$ {\text{SO}}_{4}^{{{ } - }} \cdot { } + {\text{ S}}_{2} {\text{O}}_{8}^{{{ }2 - }} \to {\text{ S}}_{2} {\text{O}}_{8}^{{{ } - }} \cdot { } + {\text{ SO}}_{4}^{{{ }2 - }} $$

4 $$ {\text{OH}} \cdot { } + {\text{ S}}_{2} {\text{O}}_{8}^{{{ }2 - }} \to {\text{ S}}_{2} {\text{O}}_{8}^{{{ } - }} \cdot { } + {\text{ OH}}^{ - } $$

The effect of PMS dosage was evaluated within the range of 1.0–4.0 g/L (Fig. 2b). In this test, we also conducted a urea degradation test in the PMS only system. The highest urea removal efficiency was achieved in the PMS + PAC system with a PMS dosage of 2.0 g/L, which was significantly higher than the PMS only system. These results indicate that the PAC is an effective catalyst for PMS activation. However, a slight decrease in the urea removal efficiency (96.6%) was observed in the PMS + PAC system at the PMS dosage of 4.0 g/L. This decrease can be attributed to the excessive PMS dosage reached an inhibition to scavenge free radicals^35^. In addition, the PMS degradation efficiency was also evaluated from the measurement of PMS concentration (Fig. 2c). In the PMS system, only a small amount of PMS was consumed (5.0%). In contrast, in the PMS + PAC system, the PMS consumption efficiency reached 98.9% at a dosage of 0.5 g/L, indicating that most of the PMS was transformed into ROS. However, as the PMS dosage increased to 4.0 g/L, the PMS consumption efficiency decreased to 50.1%. These results suggest that as the PMS dosage increases, the amount of PMS consumed by the fixed dosage of PAC remains constant.

**Figure 3:**
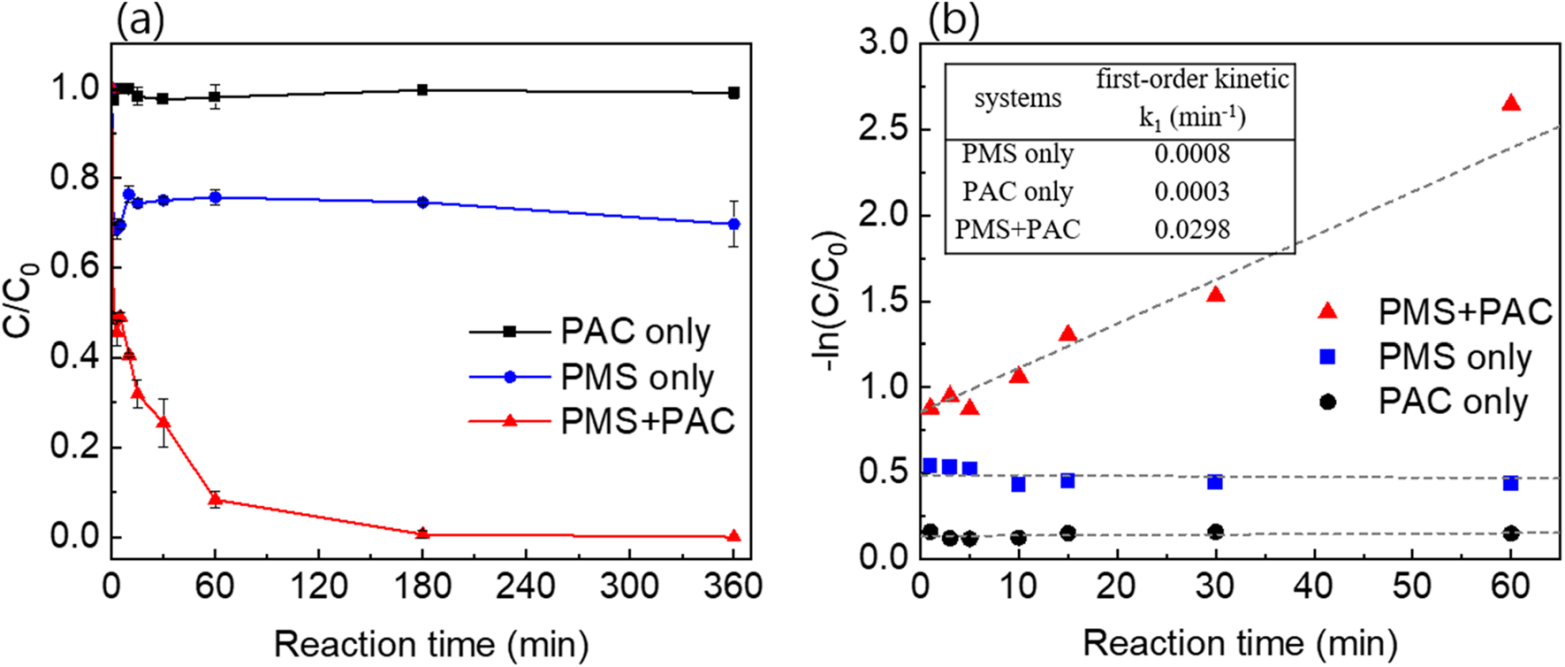
(**a**) The effect of reaction time for urea degradation in various systems and (**b**) first-order kinetic model.

As illustrated in Fig. 3a, the urea degradation kinetics in PAC, PMS, and the PMS + PAC systems were investigated. In the PAC only system, the urea concentration remained almost constant with no significant decrease observed. This result indicates that PAC could not remove urea via the adsorption mechanism alone without oxidizing agents. In the PMS only system, approximately 30% of urea was rapidly degraded within 1 min, but further degradation did not occur. We analyzed the PMS concentration during this reaction using the KI photometric method, revealing that most of the PMS remained in the solution without decomposition (Fig. S1a). This suggests that PMS was not conversed to ROS. Thus, it can be concluded that about 30% of urea removal by PMS system is attributed to its own oxidation power without conversion to ROS. In contrast, in the PMS + PAC system, the urea concentration gradually decreased with increasing reaction time, and complete removal of urea (100%) was achieved within 3 h. Additionally, it was observed that PMS was consumed almost halfway as the reaction time increased. This implies the ROSs are produced from PMS through reactions with PAC (Figure S1(b)). The kinetics of the reaction were analyzed using a first-order kinetic model, and the reaction rate constants (*k*) for each system were determined (see inset table in Fig. 3b). The first-order reaction rate constant (*k* = 0.0298 min^−1^) in the PMS + PAC system was significantly higher compared to that of the PAC only system (*k* = 0.0003 min^−1^) and the PMS only system (*k* = 0.0008 min^−1^). This indicates that the PAC, acting as a catalyst, enhances the speed and efficiency of the urea degradation reaction.

**Figure 4:**
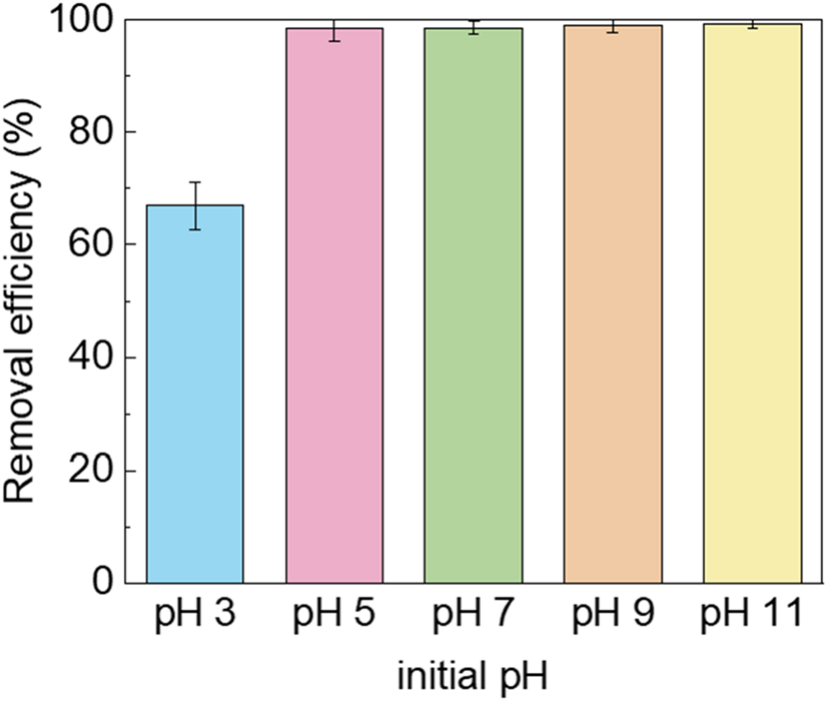
The effect of the initial pH of urea solution.

To evaluate the influence of pH on urea removal in the PMS + PAC system, the pH of the urea solution was controlled from pH 3 to 11. As depicted in Fig. 4, urea degradation was inhibited at pH 3. This can be attributed to protons binding to the more electronegative peroxide bond (O–O) of the PMS molecule under acidic conditions. This binding weakens interfacial repulsion and diminishes the catalytic performance of the system^36^. At pH 5, the concentration of H^+^ decreases, resulting in a weakened binding with the peroxide bond of PMS. Consequently, the interaction between PMS and PAC becomes more active, promoting the generation of ROS. A similar result was reported by You et al^37^. The degradation of ciprofloxacin was conducted using SR-AOP, but degradation reaction was inhibited at pH < 3. Conversely, in the PMS + PAC system, urea degradation was effective and resulted in approximately 100% removal efficiency at pH > 3. This suggests that urea removal in this system can be achieved over a wide pH range.

We also applied the urea degradation kinetic model at pH 3, pH 6, and pH 11, presenting the results in Fig. S2. As shown in Fig. S2, at pH 3, the urea degradation rate was significantly slower compared to higher pH values (*k* = 0.0091 min^−1^). As the solution pH increased to 6, the urea degradation rate rapidly accelerated, with an almost five time higher rate constant observed at *k* = 0.0465 min^−1^ within 30 min. Subsequently, the urea degradation slowed down, reaching equilibrium, and achieving a 91.1% removal at 60 min. Finally, at pH 11, the fastest urea degradation was observed among the tested pH levels, with a high reaction rate constant of *k* = 0.0979 min^−1^ obtained within 15 min. although similar urea degradation efficiency (~ 99%) was attained at pH levels exceeding 5, the speed of urea degradation is closely associated with solution pH. Faster urea degradation is achieved at higher solution pH, such as 13. After the urea degradation process, a rapid decrease in the final pH was observed in all samples, reaching approximately 2.4. This acidification of the urea solution can be attributed to the release of H^+^ from the activated PMS (Eq. 5)^38^. To mitigate the effect of acidification on the UPW system, it is necessary to use a low dosage of PMS and regularly adjust the pH.

5 $$ HSO_{5}^{ - } \to SO_{5}^{ 2 - } + H^{ + } $$

**Figure 5:**
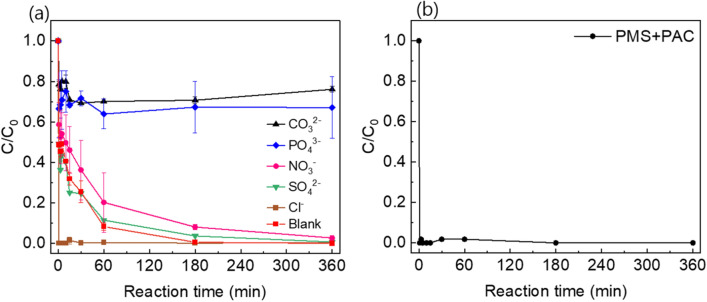
(**a**) The effect of co-existing ions and (**b**) urea degradation in reclaimed water in the PMS + PAC system.

Inorganic ions are ubiquitous in water matrices and several studies have shown that inorganic anions could affect on SR-AOP. Thus, the influence of co-existing ions (CO_3_^2−^, PO_4_^3−^, NO_3_^−^, SO_4_^2−^, and Cl^−^) on urea removal was evaluated (Fig. 5a). The influence of SO_4_^2−^ was negligible but NO_3_^−^ slightly inhibited at the beginning of the reaction. This inhibition may be attributed to the formation of nitrate radicals (NO_3_·), which have a low redox potential (2.30 V, *k* = 3.6 $\times$ 10^5^).^34^ In contrast, the presence of CO_3_^2−^ significantly inhibited urea removal. When CO_3_^2−^ co-exists, the carbonate radicals (CO_3_^−^·) which have a low redox potential (1.63 V) could be formed. Also, carbonate radicals can scavenge sulfate radicals (*k* = 6.1 $\times$ 10^6^) and halogen radicals (*k* = 8.0 $\times$ 10^7^) at a high reaction rate^39^. This leads to a decrease in the efficiency of urea degradation. Additionally, the presence of PO_4_^3−^ was shown to inhibit urea degradation. This inhibition may be attributed to PO_4_^3−^ replacing hydroxyl groups on the catalyst surface, thereby reducing the catalytic efficiency. Previous studies have also reported the inhibitory effect of PO_4_^3−^ on catalytic reactions^40^.

Interestingly, in this study, the presence of Cl^−^ dramatically accelerated the reaction of urea removal, resulting in complete degradation within 1 min. The accelerated reaction in the presence of Cl^−^ can be explained by the synergistic degradation of urea by reactive chlorine species (RCS) such as hypochlorous acid (HOCl, E^0^≒1.48 V), chlorine radical (Cl∙, E^0^ = 2.43 V), dichloride radical (Cl_2_^−^∙, E^0^ = 2.13 V), and hydroxidochlorate radical (ClOH^−^·, E^0^≒2.74 V). The asymmetric peroxide bond of PMS makes it susceptible to attack and activation by Cl^−^, leading to nucleophilic addition reactions (Eqs. 6–8)^41^. Similar results were reported by Ma et al. and Tan et al. confirming an increase in the degradation rate of saccharin and acetaminophen when low concentrations of Cl^−^ coexisted in the UV/PS system, respectively^34,42^.

6 $$ Cl^{ - } { } + { }HS{\text{O}}_{5}^{{{ } - }} { } \to {\text{ SO}}_{4}^{{{ }2 - }} + HOCl $$

7 $$ ClOH^{ - } \cdot { } \to { } \cdot {\text{OH }} + Cl^{ - } $$

8 $$ Cl^{ - } + ClOH^{ - } \cdot \to Cl_{2}^{ - } \cdot + OH^{ - } $$

Finally, we evaluated the degradation performance of the PMS + PAC system in actual reclaimed water to confirm the feasibility of the system (Fig. 5b). The reclaimed water contained various inorganic anions such as F^−^, Cl^−^, SO_4_^2−^, and NO_3_^−^. The concentrations of these ions, as well as the urea concentration, are provided in Table S1. It was observed that the PMS + PAC system achieved a 100% removal efficiency of urea within 1 min, under the same conditions as in the previous experiments. Since the concentration of inorganic anions in the actual reclaimed water was less than 10.0 mg/L, it can be considered that the influence of these ions on the urea removal process was negligible. Furthermore, the low concentration of urea and the presence of Cl^−^ may have contributed to the enhanced reaction rate of urea degradation. The pH of the reclaimed water is 6.7, and in this pH the urea degradation by PMS + PAC system is favored. Based on the above results, it can be concluded that the PMS + PAC system is highly feasible for effectively removing urea from reclaimed water to produce UPW. The system demonstrates the promising potential for practical applications in water treatment processes, particularly in the context of reclaiming water resources and ensuring the production of high-quality UPW.

### Urea degradation mechanism by PAC assisted SR-AOP

**Figure 6:**
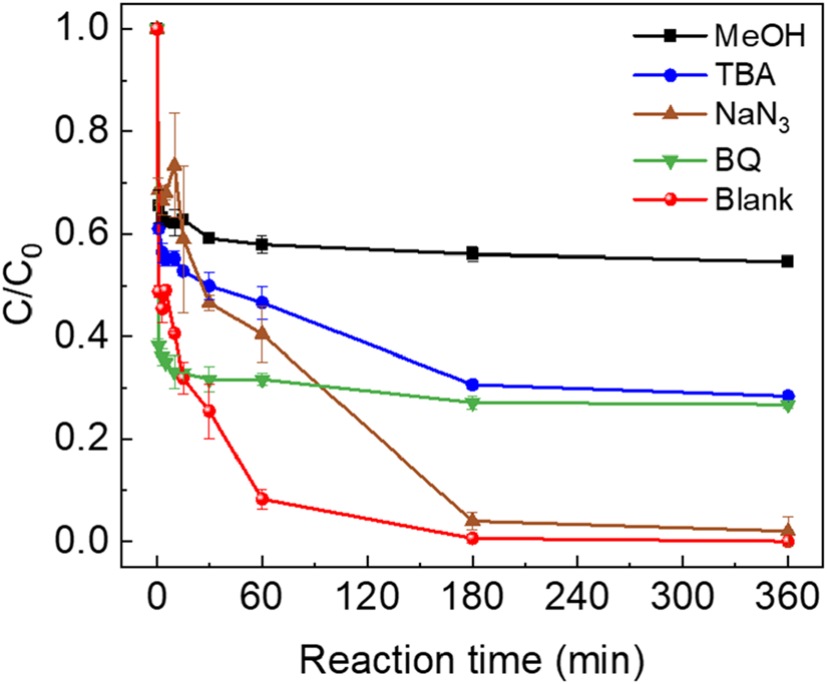
The quenching test of reactive oxygen species by various scavengers.

Figure 6 illustrated the quenching test of urea using scavengers such as MeOH, TBA, NaN_3_, and BQ to determine the roles of ROS in the PMS + PAC system. MeOH was used to quench hydroxyl radicals (·OH, *k* = (1.2–2.8)$\times$ 10^9^ M^−1^ s^−1^) and sulfate radicals (SO_4_^−^, *k* = (1.6–7.7)$\times$ 10^7^ M^−1^ s^−1^)^43^. TBA could strongly scavenge hydroxyl radicals (·OH, *k* = (3.8–7.6)$\times$ 10^8^ M^−1^ s^−1^) and NaN_3_ could scavenge single oxygens (^1^O_2_, *k* = 1.0 $\times$ 10^9^ M^−1^ s^−1^)^44,45^. BQ was used as a scavenger of superoxide radicals (O_2_^−^·, *k* = (0.9–1.9)$\times$ 10^9^ M^−1^ s^−1^)^21^. The presence of MeOH, TBA and BQ significantly inhibit degradation of urea. These results indicate that ·OH, SO_4_^−^·, and O_2_^−^·play significant roles in the degradation of urea in the PMS + PAC system. The relative contributions of these free radicals accounted for approximately 28.4% (·OH), 26.8% (O_2_^−^·), and 26.2% (SO_4_^−^·). On the other hand, when NaN_3_ was used to quench ^1^O_2_, the urea degradation reaction was retarded but it did not affect total degradation efficiency. This suggests that ^1^O_2_ is the dominant ROS at the initial stage of urea degradation but is rapidly transformed into other inactive species. This is further confirmed by the ESR analysis results presented in Figure S3. As shown in Fig. S3a, characteristic peaks of O_2_^−^∙ (six lines) and ∙OH (1:2:2:1) were observed when using DMPO as a trapping agent^46,47^. However, the peak corresponding to SO_4_^−^∙ was not detected due to nucleophilic substitution resulting in the rapid conversion of DMPO-SO_4_^−^· to DMPO-·OH^48^. Additionally, when TEMP was used as a trapping agent, characteristic peaks of TEMP-^1^O_2_ (1:1:1) were also observed (Fig. S3b)^49^. Through this analysis, we demonstrated the contributions of O_2_^−^∙, ∙OH, and ^1^O_2_ in the early stages of urea degradation in the PMS + PAC system. In summary, the relative contributions of ·OH, O_2_^−^·, and SO_4_^−^· were similar, accounting for a total of 81.4% of the urea degradation through the radical pathway. The remaining 18.6% may be attributed to non-radical pathways, such as direct electron transfer and direct oxidation by PMS. These results indicate that both radical and non-radical pathways contribute to the overall urea degradation in the PMS + PAC system.

To investigate the surface chemical structure of the PAC that contributes to PMS activation, Raman and FTIR analyses were conducted. In Raman analysis (Fig. S4), the intensity of the D peak, which represents the defective sites in the PAC's graphitic structure, noticeably decreased after urea degradation. Additionally, the I_D_/I_G_ ratio decreased from 1.709 to 0.975, indicating a decrease in the degree of defects in the PAC. These results suggest that the defective sites in the PAC act as catalysts and contribute to the activation of PMS. FTIR spectra of the PAC before and after urea degradation were presented in Fig. S5. The crucial peaks associated with CaCO_3_ (at 870 and 1407 cm^−1^) disappeared, indicating the elution of CaCO_3_ due to increased solubility caused by the lowered pH after degradation^50^. Furthermore, the peaks related to the C-O and -OH groups became wider after degradation. The broadening of these peaks suggests a decrease in their intensity, indicating a reduction in the amount of these groups. This implies that the C-O and -OH groups might have been consumed as reactive structures for PMS activation.

**Figure 7:**
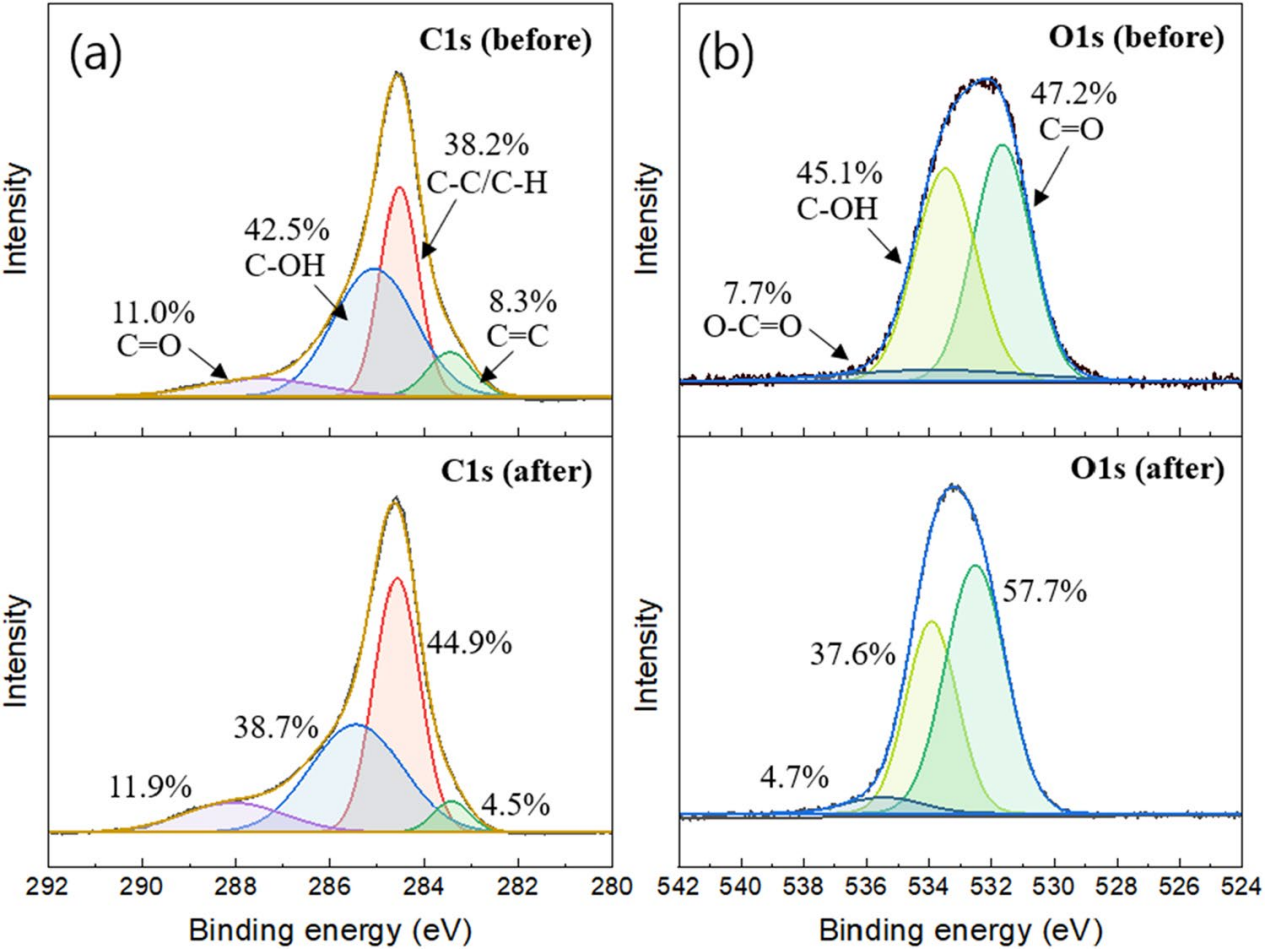
XPS spectra of (**a**) C1s, and (**b**) O1s of the PAC before and after urea degradation.

The surface chemical compositions of the PAC were analyzed by XPS (Fig. 7). The C1s spectra in Fig. 7a exhibited various functional groups of the PAC before and after urea degradation. The PAC consisted of primary compounds centering at 283.5, 284.6, 285.0, and 287.5 eV, which were attributed to C=C, C–C/C–H, C–OH, and C=O, respectively^51^. The ratio of C–OH in the pristine PAC was 42.5%, while in the used PAC, it decreased to 38.7%. This result indicated that C–OH groups could have contributed to PMS activation for the formation of ROS. As shown in Fig. 7b, the O1s spectra could be fitted into three peaks, including C=O (531.6 eV), C–OH (533.5 eV), and O–C = O (535.3 eV)^52^. Apparently, the ratios of C–OH and O–C=O in the pristine PAC were 45.1% and 7.7%, but they decreased to 37.6% and 4.7% after urea degradation. These results were almost consistent with the above results analyzed in the C1s spectra and proved that C–OH and O–C=O groups are major components contributing to PMS activation.

Based on the results above, we identified the radical species (∙OH, SO_4_^−^∙, O_2_^−^∙) contributing to urea degradation in the PMS + PAC system and determined their relative contributions. Furthermore, by observing the physicochemical changes of the PAC before and after urea degradation, we elucidated the active sites. Consequently, it can be concluded that the PAC, acting as a catalyst, can facilitate the removal of urea through two pathways: the radical pathway and the non-radical pathway. The radical pathway, which is the major process, involves the activation of PMS through the participation of oxygen-containing functional groups such as C–OH and O–C=O. This activation leads to the formation of ROS that is responsible for the degradation of urea.

## Discussion

In this study, the powdered activated carbon (PAC) assisted sulfate radical-based advanced oxidation process (SR-AOP) was investigated to remove urea from reclaimed water for ultrapure water (UPW) production. Through physicochemical analysis, it was confirmed that PAC possesses diverse functional groups and a wide specific surface area (457.8 m^2^/g), making it a promising catalyst. The urea adsorption by PAC was only < 5.0%. However, PAC significantly facilitated PMS activation in the PMS + PAC system, leading to the complete decomposition of urea in water. The reaction rate constants (*k*) of PAC, PMS, and PMS + PAC systems are 0.0003, 0.0008, and 0.0298 min^−1^, respectively. The increase in PMS and PAC dosage leads to an increase in urea removal efficiency. However, an overdose of PMS (> 4.0 g/L) and PAC (> 0.4 g/L) results in a decreasing tendency due to the termination reaction of free radicals. The PMS + PAC system demonstrated stable urea removal over a wide pH range of pH ≥ 5. The presence of CO_3_^2−^ and PO_4_^3−^ in the water matrix inhibited urea removal, while the coexistence of Cl^−^ promoted urea removal through reactive chlorine species. Furthermore, the system successfully achieved 100% urea removal in reclaimed water, making it applicable for UPW production. To elucidate the urea removal mechanism, quenching tests and analysis of physicochemical properties of PAC after degradation were performed. This study confirmed the relative contribution SO_4_^−^·, ·OH, and O_2_^−^· to the radical pathway in urea removal (81.4%). The defect sites and oxygen-containing functional groups (C–OH and O–C=O) of PAC could accelerate PMS activation. In summary, the PMS + PAC system has been demonstrated to be a promising process for urea removal in reclaimed water. These outcomes will make a significant contribution to the development of catalysts for advanced oxidation and UPW production, and it is also necessary to test various carbon-based materials as catalysts.

## Methods

### Materials

Urea (CO(NH_2_)_2_, 99.0%), peroxymonosulfate (PMS, KHSO_5_·0.5KHSO_4_·0.5K_2_SO_4_), 2,3-butanedione monoxime (C_4_H_7_NO_2_, 99.0%), antipyrine (C_11_H_12_N_2_O) and sodium bicarbonate (NaHCO_3_, 99.7%) were obtained from Sigma-Aldrich (USA). 5,5-dimethyl-1-pyrroline N-oxide (DMPO, C_6_H_11_NO, 97.0%), and 2,2,6,6-tetramethyhlpiperidine (TEMP, C_9_H_19_N, 98.0%) were obtained from TCI (Japan). Powdered activated carbon (PAC), sodium nitrate (NaNO_3_, 99.0%), sodium sulfate (Na_2_SO_4_, 99.0%), sodium carbonate (Na_2_CO_3_, 99.0%), sodium phosphate (NaH_2_PO_4_, 99.0%), methyl alcohol (MeOH, CH_3_OH, 99.5%), tert-butyl alcohol (TBA, (CH_3_)_3_COH, 99.5%), sodium azide (NaN_3_, 99.0%), 1,4-benzoquinone (BQ, C_6_H_4_O_2_), acetic acid (CH_3_COOH, 99.5%) and potassium iodide (KI, 99.5%) were obtained from Daejung (Republic of Korea). Sodium hydroxide (NaOH, 98.0%) and sodium chloride (NaCl, 99.0%) were obtained from Samchun (Republic of Korea). Also, reclaimed water was obtained from Cheong-ju wastewater treatment plant (Republic of Korea) in August 2023.

### Characterization of PAC

The degree of graphitization and defects of PAC was analyzed by Raman spectroscopy (Nanophoton, RAMANtouch). Also, crystalline structure and surface groups were analyzed by X-ray diffraction spectrometer (XRD, Bruker, D8 Discover) and Fourier transform infrared spectrometer (FTIR, Agilent, Cary 670). Specific surface area and pore size were analyzed by the N2 adsorption–desorption method by BET analyzer (Protech, ASAP 2020). The valence state of elements existing on the PAC was analyzed by X-ray photoelectron spectroscopy (XPS, Quantera-II, PHI). The identification of reactive oxygen species was conducted through Electron spin resonance (ESR, Jeol, JES-FA100).

### Urea degradation test

The degradation performance was evaluated in a batch reactor with 250 mL urea solution (5.0 mg/L). The desired amount of PMS (2.0 g/L) and PAC (0.2 g/L) was added to the urea solution. The mixture was stirred for 6 h at 500 rpm and 25 °C. After urea degradation, the solution was filtered using a 0.45 μm syringe filter. Then, the final concentration of urea was determined using a UV–Vis spectrophotometer (Shimadzu, UV-1280). All the tests were done in duplicate, and the data was expressed as the mean values.

The urea degradation kinetic was evaluated. The solution was taken at 1, 3, 5, 10, 15, 30, 60, 180, and 360 min to measure the concentration of urea in the solution. The initial pH of the solution was controlled by various values (3, 5, 7, 9, and 11) adding 0.1 M and 1.0 M NaOH and HCl solutions. Also, to evaluate the degradation efficiency of urea in the water matrix, 5.0 mM inorganic anions (Cl^−^, NO_3_^−^, CO_3_^2−^, SO_4_^2−^, and PO_4_^3−^) were added to the urea solution. To identify the reactive oxygen species (ROS), methanol, tert-butyl alcohol, sodium azide, and 1,4-benzoquinone were used to quench the ROS as scavengers. The analysis of the sample using ESR was conducted at the initial stage of urea degradation. After the reaction initiation, trapping agents (DMPO, TEMP) were injected 5 min later, followed by a stabilization period of 2 min before measurement. Furthermore, the urea degradation performance was also evaluated in reclaimed water. The compositions of reclaimed water are shown in Table S1. The concentration of the compositions was analyzed by inductively coupled plasma optical emission spectrometer (ICP-OES, Agilent, 5800) and ion chromatography (IC, Dionex, ICS-4000).

### Analytical methods

The diacetyl-monoxime method (DAMO) was used as an analysis method of urea^19^. Specifically, 10.0 mL urea sample, 1.0 mL diacetyl-monoxime (2%), and 2.0 mL antipyrine (0.2%) were diluted with 25.0 mL deionized water in an amber tube. The sample was mixed using a vortex mixer, put into a boiling water bath for 50 min, and then cooled. The concentration of urea was measured by UV–Vis at 460.0 nm.

The KI spectrophotometric method was used as an analysis method of PMS^53^. 1.0 mL PMS sample, 1.0 g KI, 0.2 g NaHCO_3_ and 49.0 mL deionized water were taken in a 50.0 mL tube. The sample was mixed using a vortex mixer and then kept for 10 min to achieve the sample’s stability. The concentration of PMS was measured by UV–Vis at 352.0 nm.

### Supplementary Information

Supplementary Information.

## Data Availability

The datasets used and/or analyzed during the current study are available from the corresponding author on reasonable request.
