# Supplementary material for: Powdered activated carbon (PAC)-assisted peroxymonosulfate activation for efficient urea elimination in ultrapure water production from reclaimed water
Source: Sci Rep. 2024 Feb 26;14:4597. doi: 10.1038/s41598-024-55414-w (PMC10897149; doi:10.1038/s41598-024-55414-w)
Supplement: Supplementary file 1 — Supplementary Information. [file 41598_2024_55414_MOESM1_ESM.docx]

*Supporting information*

Powdered Activated Carbon (PAC)-Assisted Peroxymonosulfate Activation for Efficient Urea elimination in Ultrapure water Production from Reclaimed Water

Chaelin Kim, Heeji Yoo, Gyubin Lee, Hye-Jin Hong^*^

*Department of Environmental Engineering, Chungbuk National University, Chungdae-ro 1, Seowon-Gu, Cheongju, Chungbuk 28644, Republic of Korea*

***Corresponding author**

**Prof. Hye-Jin Hong**

Tel: +82-43-261-2469; fax: +82-43-264-2465. e-mail: [hyejiny@chungbuk.ac.kr](mailto:hyejiny@chungbuk.ac.kr)

**Table S1.** Compositions of reclaimed water

|  | Components [mg/L] | | | | | | | | | |
| --- | --- | --- | --- | --- | --- | --- | --- | --- | --- | --- |
|  | Cations | | | | | Anions | | | | Urea |
|  | Ca^2+^ | K^+^ | Mg^2+^ | Na^+^ | Si^4+^ | F^-^ | Cl^-^ | SO_4_^2-^ | NO_3_^-^ |  |
| Reclaimed water | 16.6 | 15.0 | 4.60 | 26.0 | 2.60 | 0.02 | 8.60 | 3.00 | 3.00 | 0.889 |

**Table S2.** Comparisons of the catalytic ability of various carbon-based catalysts [Urea conc.: 5 mg/L, PMS: 2.0 g/L, carbon-based catalyst: 0.2 g/L]

|  | I_D_/I_G_ ratio | BET surface area (m^2^/g) | Urea removal efficiency (%) at PMS+carbon based catalyst system |
| --- | --- | --- | --- |
| PAC | 1.709 | 457.8 | 100 |
| GAC | 1.130 | 793.4 | 0.0 |
| GO | 0.860 | 0.422 | 65.8 |
| SWCNT | 0.044 | 508.3 | 0.0 |
| MWCNT | 0.818 | 191.5 | 65.2 |


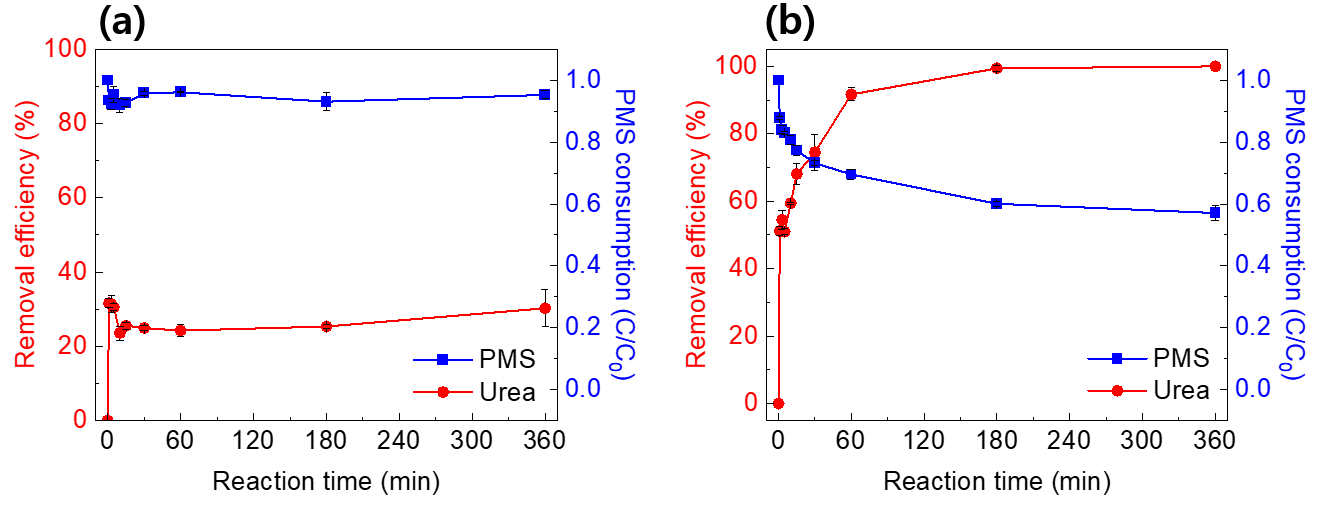


**Figure S1.** The removal efficiency of urea and PMS consumption (a) in the PMS only and (b) PMS+PAC system


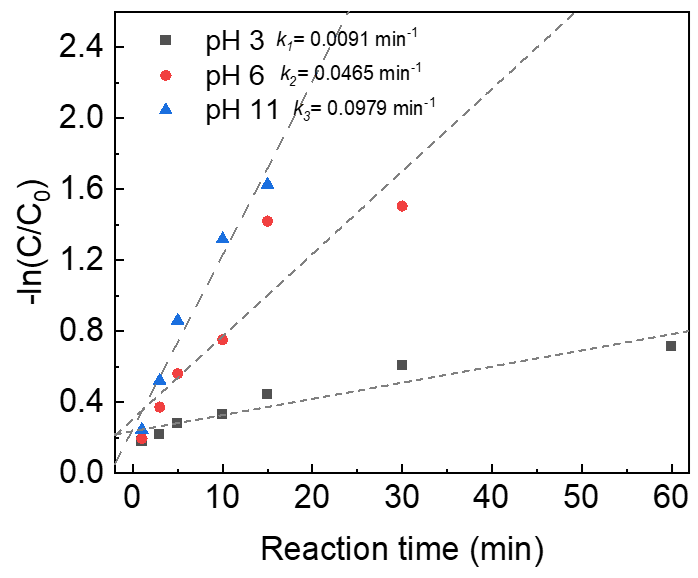


**Figure S2.** Urea degradation kinetic of PMS+PAC system at pH 3, pH 6, and pH 11

[Urea: 5 mg/L, PMS: 2.0 g/L, PAC: 0.2 g/L]


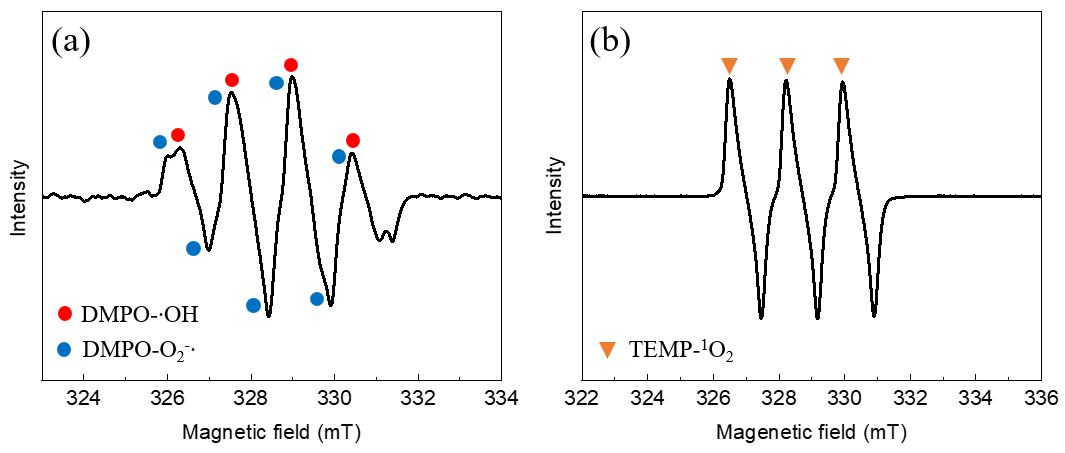


**Figure S3.** The ESR spectrum of (a) DMPO-O_2_^-^∙, DMPO-∙OH, and (b) TEMP-^1^O_2_ in the PMS+PAC system

**
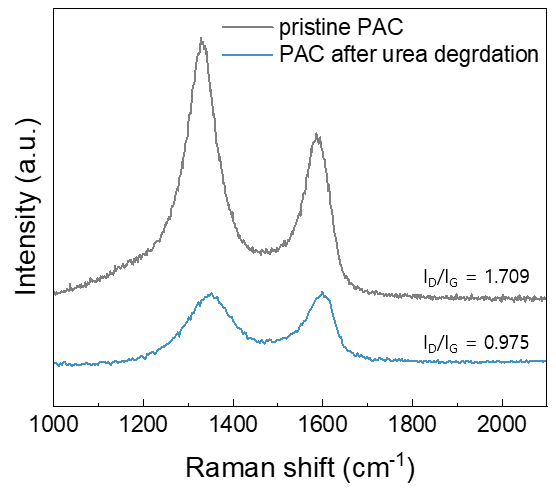
**

**Figure S4.** Raman spectra of the PAC before and after urea degradation


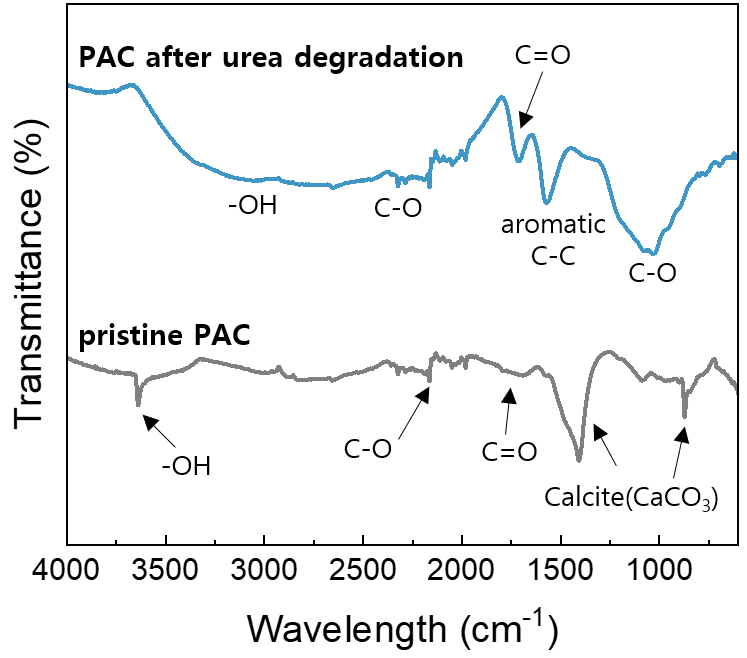


**Figure S5.** FTIR spectra of the PAC before and after urea degradation

**[Reaction Kinetic Models]**

First-order kinetic model can be written as:

$\ln\left[ C \right]= -kt + {[C]}_{0}$ (1)

Where *C_0_* (mg/L) denotes the initial concentration of urea solution, and *C* (mg/L) is the final concentration after degradation. *k* is the reaction rate constant of first-order and second-order kinetic models.
